# Mass spectrometry-based identification of new serum biomarkers in patients with latent infection pulmonary tuberculosis

**DOI:** 10.1097/MD.0000000000032153

**Published:** 2022-12-02

**Authors:** Yan-Xia Li, Kang-Di Zheng, Yu Duan, Hua-Juan Liu, Yu-Qun Tang, Jun Wu, Dong-Zi Lin, Zhao Zhang

**Affiliations:** a Department of Laboratory Medicine, Foshan Forth People’s Hospital, Foshan, Guangdong, China; b Research and Development Department, Foshan Longsee Biotechnology Co, Ltd., Foshan, Guangdong, China; c Department of Respiratory Medicine, Affiliated Hospital of Guangdong Medical University, Zhanjiang, Guangdong, China; d Department of Laboratory Medicine, Fourth People’s Hospital of Nanhai District of Foshan City, Foshan, Guangdong, China; e Peptide and Protein Research and Application Key Laboratory of Guangdong Medical University, Zhanjiang, Guangdong, China.

**Keywords:** latent infection, LC-MS/MS, serum biomarker, tuberculosis

## Abstract

Noninvasive and simple indicators for diagnosing latent tuberculosis (TB) infection (LTBI) and tracking progression from latent infection to active TB infection are still desperately needed. The aim of this study was to screen and identify possible biomarkers for diagnosing LTBI and monitoring the progression from latent infection to active TB infection, as well as to investigate the underlying processes and functions. To assess changes in metabolite composition associated with active tuberculosis infection in humans, whole blood supernatants were collected from patients with LTBI, drug-susceptible TB patients, drug-resistant TB patients, and healthy controls. The metabolites in all serum samples were extracted by oscillatory, deproteinization, and then detected by liquid chromatography-tandem mass spectrometry/MS analysis. Normalization by Pareto-scaling method, the difference analysis was carried out by Metaboanalyst 4.0 software, and 1-way analysis of variance analysis among groups showed that *P*-value < 0.05 was regarded as a different metabolite. To clarify the dynamic changes and functions of differential metabolites with disease progression, and explore its significance and mechanism as a marker by further cluster analysis, functional enrichment analysis, and relative content change analysis of differential metabolites. 65 metabolites were substantially different in four groups. Differential metabolites such as Inosine and Prostaglandin E1 may be important blood indicators for diagnosing *mycobacterium tuberculosis* latent infection, which were all tightly connected to amino acid metabolism, Biosynthesis of various secondary metabolites, Nucleotide metabolism, Endocrine system, Immune system, Lipid metabolism, and Nervous system. This study screened and identified Inosine, 16, 16-dimethyl-6-keto Prostaglandin E1, Theophylline, and Cotinine as potential serum biomarkers for diagnosing latent TB infection, and Cotinine as potential biomarkers for monitoring disease progression from healthy population to LTBI and then to active TB including drug-resistant TB infection and sensitive TB infection. Furthermore, this research provides a preliminary experimental basis to further investigate the development of metabolomics-based diagnosis of LTBI and monitoring the progress from latent infection to active TB infection.

## 1. Introduction

Tuberculosis (TB) is mainly caused by *Mycobacterium tuberculosis* (*M. tuberculosis*).^[1]^ Every year, 9.4 million people worldwide are diagnosed with tuberculosis, and more than 1.3 million die from it. TB has emerged as a serious worldwide health threat. Because the culture of *M. tuberculosis* is difficult and time-consuming, the number of patients with TB latent infection is huge. At present, 2 main immune-based approaches are currently used for identification of latent tuberculosis infection (LTBI): the tuberculin skin test (TST) and the interferon-gamma release assay (IGRA). Unfortunately, neither test can accurately differentiate between LTBI and active TB, and the reduced sensitivity in immunocompromised patients, which were cited as some of the major drawbacks of the current LTBI tests by the expert panel.^[2]^ Cohort studies have shown that both TST and IGRA have low predictive value for progression from infection to active TB.^[3]^ Therefore, the panel emphasized the importance of research to develop better point-of-care diagnostic tests and novel biomarkers able to predict the progression from LTBI to active TB.

Small molecule metabolites are the end products of the cell regulation process. Changes in their kinds and amounts are regarded as organisms’ ultimate reactions to a gene or environmental changes.^[4,5]^ Gas chromatography-mass spectrometry, liquid chromatography-tandem mass spectrometry (LC-MS), nuclear magnetic resonance, and other metabolomics detection technologies with high flux and high sensitivity are the mainstream methods for metabolite detection.^[6]^ So far, metabonomics technology has been widely used in clinical research, such as the identification of new biomarkers and how biomarkers help to improve the discovery and diagnosis of diseases.^[7–9]^

The discovery of TB-related metabolites has also brought new methods for tuberculosis diagnosis and therapy. Lau SKP *et al*^[10]^ reported that the content of 24 metabolites in the culture supernatant of *M. tuberculosis* was significantly higher than that of non-*M. tuberculosis* group, of which 4 metabolites were identified as 1-tuberculin adenosine derivatives, which may be used as a new marker of metabolism of *M. tuberculosis*. Yeware A *et al*^[11]^ focused on the mechanism of rapid transition of *M. tuberculosis* from the active state to the viable but non-culturable state caused by ammonium diphenylene alkylate treatment through metabolomics and compared it with the dormant phenotype.

The above studies indicated that new metabolic markers in *M. tuberculosis*, which may be related to the progression of tuberculosis. Simultaneously, the concentration of serum metabolites is a direct reading of human biological processes, which is related to cardiovascular and metabolic diseases. Therefore, it is necessary to study the serum metabolites of tuberculosis patients and the progress of tuberculosis. Feng S *et al*^[12]^ studied the metabolic patterns of tuberculosis patients, healthy individuals, and nontuberculosis patients. The difference in serum small molecule abundance among the 3 groups was determined by comparing 3 groups. 12 metabolites were found to contribute to the distinction between the TB active group and the control groups. Zhou A *et al*^[13]^ use nuclear magnetic resonance-based metabolomics on serum samples from TB patients and healthy individuals demonstrated a total of 17 metabolites differed significantly in concentration between the 2 groups. These researches showed that the dynamic changes of metabolomics in TB patients during the treatment process were systematically analyzed, and the serum biomarkers of new latent infection and active tuberculosis were screened, and the main molecular mechanisms were explored. However, patients with latent TB infection and drug-resistant TB were not included in these studies, and thus, the potential of such a metabolic profile for monitoring tuberculosis progression remains to be determined. The purpose of this study was to screen specific metabolic markers through LC-MS/MS technology to diagnose LTBI and evaluate the development from latent infection to active tuberculosis infection, which is expected to formulate personalized treatment plans for patients.

## 2. Methods

### 2.1. Subjects and sample collection

From October 2017 to March 2018, blood samples were obtained from patients undergoing pulmonary TB screening at Foshan Forth People’s Hospital and Dongguan Sixth People’s Hospital. The criteria for enrollment of TB patients, LTBI individuals and healthy population included the following: TB patients were diagnosed based on clinical symptoms (such as fever, coughing and productive sputum), and sputum-smear-positivity and culture-positivity for *M. tuberculosis*; The TB patients had never been treated with any anti-tuberculosis (anti-TB) drug; LTBI individuals were with no clinical signs of TB with positive IGRA test; Healthy individuals with negative IGRA test; All study participants agreed to participate in the study and signed informed consent. The exclusion criteria included the following: Study participants with positive pnitrobenzoic acid (PNB) test; Study participants had hepatitis infections or autoimmune disorders and were taking any immunosuppressive medical treatment; HIV test was positive; Patients with extrapulmonary TB, malignant tumors, and chronic metabolic diseases. According to the results of sputum smear, sputum culture IGRA test, and drug susceptibility test, the collected samples were divided into groups of healthy control (HC) with 148 (56.92%), latent infection with 44 (16.92%), drug resistant with 38 (14.62%) cases, and drug sensitivity (DS) with 30 (11.54%) cases. Following the collection of blood samples, the serum was centrifuged and kept at -20°C for LC-MS/MS analysis. The ethics committees of the Dongguan Key Laboratory of Medical Bioactive Molecular Developmental and Translational Research, Foshan Fourth People’s Hospital, and Dongguan Sixth People’s Hospital all gave their approval to this study.

### 2.2. Interferon gamma (Ifn-γ) release assay (IGRA)

IGRA test detects M.tb infection by measuring immunologic responses of *T*-cells, which release IFN-*γ* following stimulation by specific M.tb antigens. The QuantiFERON-TB Gold In-Tube assay (QFT-GIT; Qiagen, Germantown, MD), an enzyme-linked immunosorbent assay (ELISA), is the most commonly used IGRA to identify LTBIs. The QFT-GIT assays were performed according to the manufacturer’s instructions. Whole blood collected in lithium-heparin tubes was distributed (with 1 mL of whole blood in each tube) into TB antigen tube (TB), mitogen tube, and nil tube for both assays. Each tube was incubated for 20 hours at 37 °C. The tubes were then centrifuged for 15 minutes at 2000 × g, and IFN-*γ* levels (IU/mL) in the plasma were measured by enzyme-linked immunosorbent assay. According to manufacturer’s guidelines, the results were considered positive when the TB-Nil value was ≥ 0.35 IU/mL and ≥ 25% of the nil value

### 2.3. Drug susceptibility test

Drug susceptibility was determined by the proportion method on Löwenstein-Jensen medium, with the following concentrations for the 2 first-line anti-TB drugs: 0.2 *μ*g/mL for isoniazid, and 40 *μ*g/mL for rifampicin. The criterion for resistance was 1% or more colonies in the drug-containing medium when compared to the number of colonies developing in the drug-free medium.

### 2.4. Preconditioning of serum

For ultrasonic processing, 100 *μ*L of serum were mixed with 600 *μ*L of methanol (containing 1 mM butylhydroxytoluene) for 5 minutes. Then, after 1 hour at room temperature, add 1.8 mL of Methyl Tertbutyl Ether. Add 500 *μ*L of water and incubate at room temperature for 10 minutes, without stopping to oscillate. After standing for 2 minutes, samples were centrifuged for 10 minutes at 12,000 rpm. Using mixed layer and aqueous layer metabolite conditions, pipette 600 *μ*L of upper layer lipid and 300 *μ*L of bottom layer aqueous layer (2:1) and transfer to a fresh EP tube, then dissolve with 200 *μ*L of acetonitrile and water (1:1) after vacuum drying, then centrifuge to remove the supernatant.

### 2.5. Metabolic profiling detection by LC-MS

An ACE (Aberdeen, Scotland) Excel2C-18PFP (100 × 2.1 mm, 2 *μ*m) chromatographic column and a C18 guard column were utilized in the experiment. Aqua with 0.1% formic acid was used in mobile phase A, while acetonitrile with 0.1% formic acid was used in mobile phase B. The chromatographic gradient began at 2% B for 1 minute and ended at 98%B after 10 minutes. This lasted for 2 minutes before dropping to 2% in 30 seconds before returning to equilibrium for 3 minutes. The injection volume was 2 *μ*L, and the column temperature was kept at 35 °C Celsius. All of the samples were given 2 injections.

With a spray voltage of 3.5 kV, a capillary temperature of 300°C, a sheath gas flow of 50, and an assist gas of 10, the mass spectrometer was operated in heated electrospray ionization (HESI) mode. The complete scan’s collecting resolution ratio was 70,000, and the MS/MS collecting list was 17,500.

### 2.6. The identification and screening of the metabolites

Using MetaboAnalyst 4.0 software, the data were normalized using the Pareto-scaling approach, and then the differences and enrichment analyses were carried out simultaneously. For univariate analysis, the statistical significance of metabolites features was determined among latent TB infection, DS TB, drug-resistant TB, and healthy controls using 1-way analysis of variance (ANOVA) by MetaboAnalyst 4.0. To further screen the differential metabolites among different groups, principal components analysis (PCA) and orthogonal partial least squares-discriminant analysis (OPLS-DA) analyses were performed, and the variable importance projection (VIP) value was used to screen potential biomarkers. The cutoff criteria for screening differentially expressed metabolites were *P* *< *.05 and VIP* > *1. The Metlin_AMRT_PCDL and Metlin_Lipids_AM_PCDL databases were used to identify the metabolites from the differential data. The samples were then subjected to a Hierarchical Clustering Analysis to predict potential serum biomarkers for the detection of latent infection and active tuberculosis utilizing the metabolites with significant differences.

### 2.7. Statistical analysis

The results were further annotated with Kyoto Encyclopedia of Genes and Genomes (KEGG), and Human Metabolome Database, and then were analyzed using modules of pathway analysis and enrichment analysis of MetaboAnalyst 4.0.^[14–16]^ One-way ANOVA was used for the comparison of metabolites to determine statistically significant differences among the 4 groups by MetaboAnalyst 4.0. A *P* value of < 0.05 was considered to be statistically significant. Furthermore, MetaboAnalyst and R packages were employed to construct statistical plots, including PCA and OPLS-DA. The VIP was used to identify potential biomarkers with a cutoff of 1.

## 3. Results

### 3.1. Clinical information of the subjects

The clinical and demographic characteristics of the recruited participants are summarized in Table 1. A total of 260 subjects were included in this study. Forty-four LTBI cases (16.92%) with known IGRA-positive results without active TB symptoms were enrolled in the latent infection group. We enrolled 38 patients (14.62%) with resistance to 2 first-line anti-TB drugs (isoniazid and rifampicin) in the drug resistan group, and 30 patients (11.54%) with susceptibility to isoniazid or rifampicin in the DS group. The HC group included 148 healthy volunteers (56.92%) without risk of TB exposure.

**Table 1:** The clinical information between healthy control, latent infection, drug resistant, drug sensitivity group (x ± s).

Indicators	healthy control (HC)	latent infection (LI)	drug resistant (DS)	drug sensitivity (DR)
Number/n	148	44	38	30
Age (median ± SD, range)	37.65 ± 8.84 (18–60)	42.34 ± 9.11 (22–57)	39.08 ± 15.19 (21–68)	39.1 ± 12.83 (19–62)
Male	78 (52.70%)	29 (65.91%)	30 (78.95%)	19 (63.33%)
Female	70 (47.30%)	15 (34.09%)	8 (21.05%)	11 (36.67%)
sputum smear/culture (positive, n)	0	0	38	30
sputum smear/culture (negative, n)	148	44	0	0
*γ* Interferon (Nil, IU/mL)	0.10 ± 0.34	0.27 ± 0.75	0.54 ± 0.48	0.06 ± 0.15
*γ* Interferon (TB-Nil, IU/ML)	-0.02 ± 0.22	10.46 ± 14.30	9.35 ± 16.21	8.82 ± 17.74
*γ* Interferon (Mitogen-NiL, IU/mL)	40.63 ± 7.12	36.86 ± 12.51	27.56 ± 26.22	28.91 ± 21.37
IGRA test (positive, n)	0	44	38	30
IGRA test (negative, n)	148	0	0	0
Drug susceptibility test (%)	/	/	38 (100%)	30 (100%)
Leukocyte count/n	6.81 ± 1.58	6.91 ± 1.65	7.92 ± 2.65	5.93 ± 2.20
Lymphocyte ratio (%)	31.85 ± 6.47	30.45 ± 6.91	27.51 ± 10.20	23.28 ± 6.42
Monocyte ratio (%)	7.44 ± 2.04	7.69 ± 1.80	8.28 ± 1.77	8.67 ± 2.46

DR = drug resistant, DS = drug sensitivity, HC = healthy control, IGRA = Interferon-gamma release assay, LI = latent infection, TB = Tuberculosis.

### 3.2. Analysis of differential metabolite distribution

**Figure 1.**
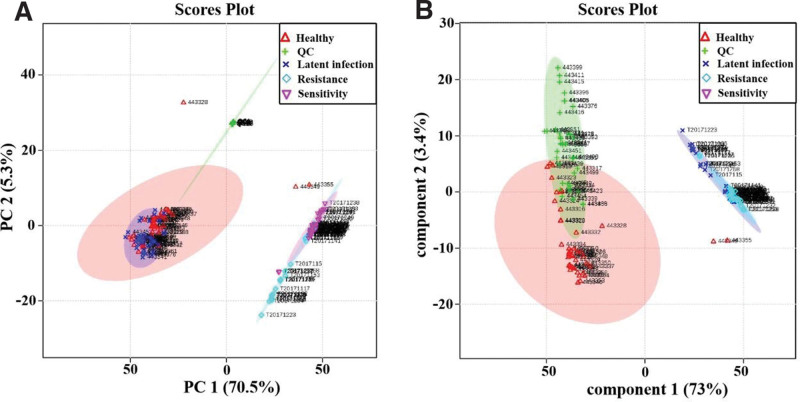
Analysis of serum metabolites in each group by PCA and OPLS-DA. (A) The PCA analysis of healthy control, latent infection, drug resistant, and drug sensitive group; (B) The OPLS-DA analysis of healthy control, latent infection, drug resistant, and drug sensitive group. OPLS-DA = partial least squares discriminant analysis, PCA = principal component analysis.

The distribution of serum metabolites in healthy control and latent infection group were analyzed by LC-MS. Before the analysis by SMICA-P13.0 software, the data was normalized to ensure more intuitive and reliable results. PCA showed that there was no significant difference between healthy control and latent infected groups (Fig. 1A). OPLS-DA could distinguish the latent infected group from the healthy control group, suggesting that there were differences in metabolite components between the 2 groups (Fig. 1B). In addition, a series of serum metabolic markers of drug-resistant and drug-sensitive groups were screened in the early stage by metabolomics technology. PCA and OPLS-DA analysis implicated that drug-resistant and drug-sensitive groups could be significantly distinguished from healthy control and latent patient groups (Fig. 1).

### 3.3. Screening differential metabolites in different groups

One-way ANOVA was used to examine the serum metabolites of individuals in the health control, latent infection, drug sensitive, and drug resistant groups. The results revealed 565 significantly different metabolites among the 4 groups (*P* < .05) (see Table S1, Supplemental Digital Content, http://links.lww.com/MD/I53, Supplemental Content, which showed the metabolites with significant difference among the 4 groups).

**Figure 2.**
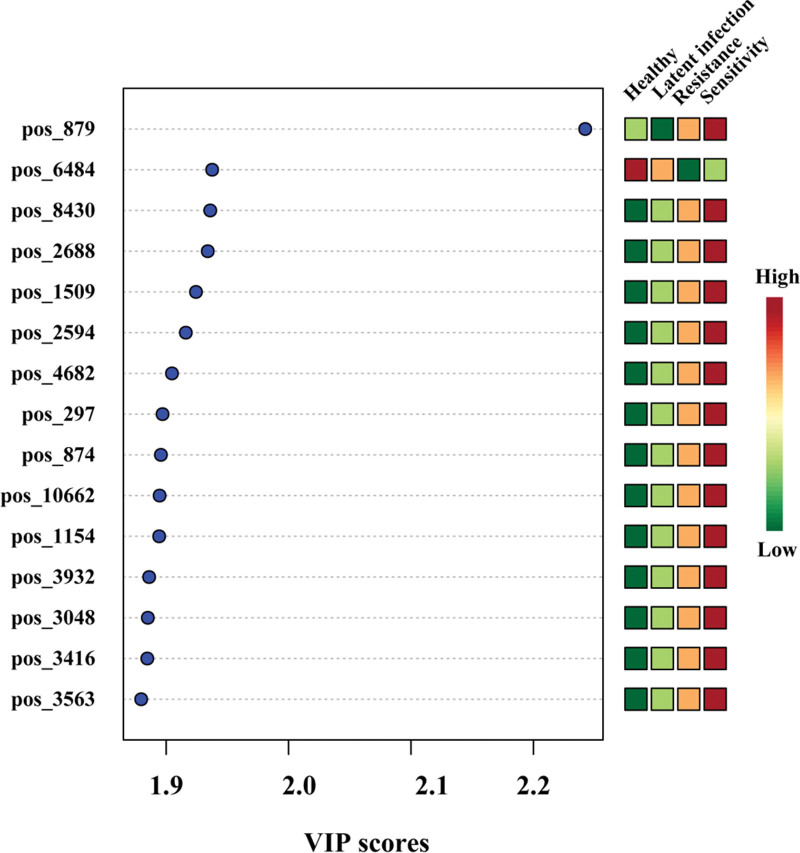
Partial least squares discriminant analysis (PLS-DA) variable importance in projection (VIP) plot of significantly differential metabolites in healthy control, latent infection, drug resistant patients, drug sensitive group. The x-axis represented the VIP scores, and the y-axis represented the compounds. Red and green colors represented increased and decreased levels of metabolites, respectively.

To further screen the differential metabolites among different groups, VIP was used to screen the key variables in the grouping based on OPLS-DA analysis. As shown in Figure 2, the VIP scores of pos_879 (Cotinine) compound was the largest, which was a significant difference metabolite in the 4 groups (see Table S2, Supplemental Digital Content, http://links.lww.com/MD/I53, Supplemental Content, which showed the metabolites with significant difference among the 4 groups), and was up-regulated in the drug sensitive group and down-regulated in the latent infection group. In addition, the results also showed that 26 compounds including POS_ 1509 (Phencyclidine), POS_2594 (Ranitidine), and POS_3048 (13E-Docosenamide) were at higher levels in the in sensitive group compared to the drug-resistant group, latent infection group and healthy control group. Therefore, they can be used as potential markers to distinguish drug-sensitive TB infection patients, including pos_3048 (13e docosenamide) a significantly different metabolite in 4 groups (see Table S1, Supplemental Digital Content, http://links.lww.com/MD/I53, Supplemental Content, which showed the metabolites with significant difference among the 4 groups).

### 3.4. Cluster analysis of serum metabolic biomarkers

**Figure 3.**
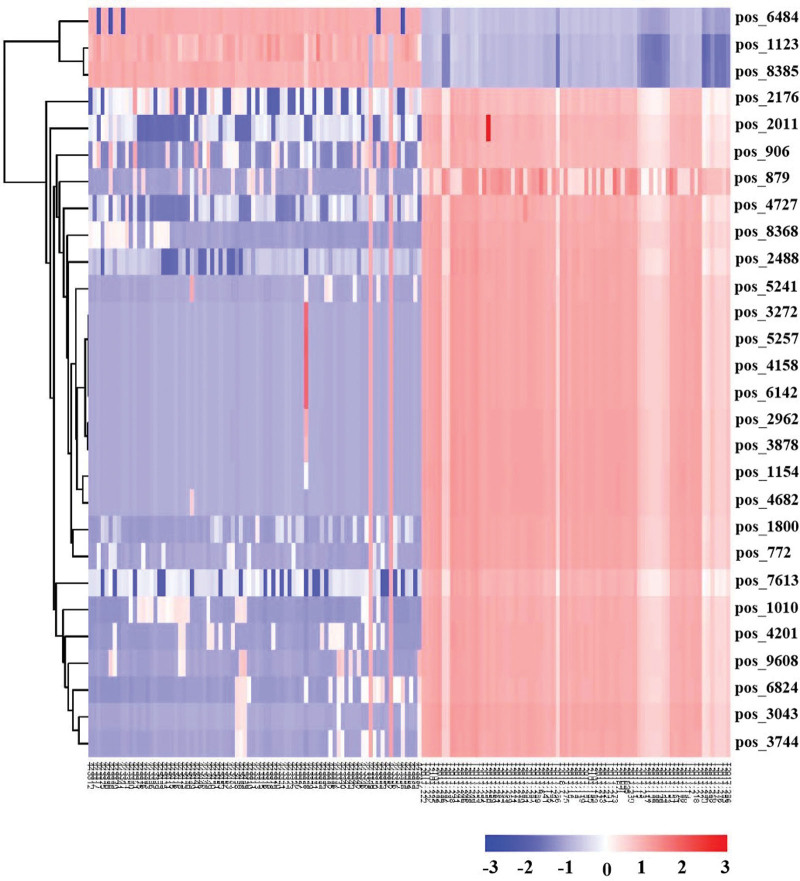
Clustering results of metabolic biomarkers in healthy control, latent infection, drug resistant, and drug sensitive group. The x-axis represented samples, and the y-axis represented the M/z of compounds.

Cluster analysis was conducted on individual differential metabolites in healthy control group, latent infection group, drug resistant group and sensitive group (Fig.3).

We found that in the healthy control and latent infection groups, pos_6484 (PC(16:0/0:0)[U]/PC(16:0/0:0)[rac]), pos_1123 (DL-Tryptophan), pos_8385 (Leu-Leu-Asp-Leu-Leu) were up-regulated, and pos_2176 [(&amp;plusmn;)-Octanoylcarnitine), pos_2011(Phe Ile), pos_906 (Theophylline), pos_879 (Cotinine), pos_4727 (Ala Asn Val Asp), pos_8368 (Phe Glu Ser Phe Gly), pos_2488(4-(1-Acetyloxypropen-2-yl-)-2-methoxyphenylisobutyrat,4-(1-Acetoxy-2-propen-1-yl)-2-methoxyphenyl 2-methylpropanoate], pos_5241 (Arg Met Met), pos_3272 (Ala Met Lys), pos_5257 (HC Toxin), pos_4158 (Asn Phe Ile), pos_6142 ((3E)-7-Hydroxy-3,7-dimethyl-3-octen-1-yl6-O-(6-deoxy-?-L-mannopyranosyl)-?-D-glucopyranoside), pos_2962 (PGH2), pos_3878 (16,16-dimethyl-6-keto Prostaglandin E1), pos_1154 (PROPOXUR), pos_4682 (His Lys Met), pos_1800 (Inosine), pos_772 (Indoleacetaldehyde), pos_7613 (Arg Thr Asp Arg), pos_1010 (3-Methylethcathinone), pos_4201 (Arg Gly Tyr), pos_9608 (Arjunglucoside II; (2S,3R,4S,5S,6R)-3,4,5-trihydroxy-6-(hydroxymethyl)tetrahydro-2H-pyran-2-yl (4aS,6aS,6bR,9R,10R,11R,12aR)-10,11-dihydroxy-9- (hydroxymethyl)-2,2,6a,6b,9,12a-hexamethyl-1,3,4,5,6,6a,6b,7,8,8a,9,10,11,12,12a,12b,13,14b-octadecahydropicene-4a(2H)-carboxylate), pos_6824 (PG(18:1(9Z)/0:0)), pos_3043 (Lisuride), pos_3744 (Spiromesifen) were down-regulated. The above metabolites differentially expressed among healthy control and latent infection groups can be used as potential markers to distinguish healthy controls and patients with latent TB infection.

### 3.5. Metabolic pathway analysis

**Figure 4.**
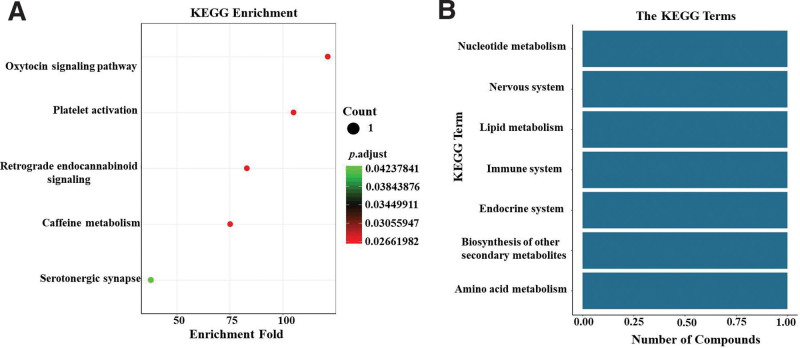
Pathway analysis of differential metabolites in healthy control, latent infection, drug resistant, and drug sensitive group. (A) The significantly enriched pathway (*p* adjust < 0.05); (B) The annotation of level 2 KEGG pathway. KEGG = Kyoto encyclopedia of genes and genes.

KEGG (Kyoto Encyclopedia of genes and genes) annotation and enrichment analysis were performed on the differential metabolites among 4 groups with *p* adjust < 0.05. As shown in Figure 4A, the significantly enriched pathway included the Oxytocin signaling pathway, Platelet activation, Retrograde endocannabinoid signaling, Serotonergic synapse, and Caffeine metabolism. And the significant compounds were Prostaglandin H2 and Theophylline. Simultaneously, the annotation of level 2 KEGG pathway included Amino acid metabolism, Biosynthesis of other secondary metabolites, Nucleotide metabolism, Endocrine system, Immune system, Lipid metabolism, and Nervous system (Fig. 4B). In addition, the significant compounds were Indole-3-acetaldehyde, Theophylline, Inosine, and Prostaglandin H2 (Table 2). Among these, Prostaglandin H2 and Theophylline were showed by enriched and annotated pathways at the same time.

**Table 2:** KEGG pathway enrichment results.

ID	Description	Count	*p* value	*p* adjust	Enrichment Fold	Compound Sig
ko00232	Caffeine metabolism	1	.01	.03	74.77	Theophylline;
ko04611	Platelet activation	1	.01	.03	104.68	Prostaglandin H2;
ko04723	Retrograde endocannabinoid signaling	1	.01	.03	82.64	Prostaglandin H2;
ko04921	Oxytocin signaling pathway	1	.01	.03	120.79	Prostaglandin H2;
ko04726	Serotonergic synapse	1	.03	.04	37.39	Prostaglandin H2;
ko00380	Tryptophan metabolism	1	.05	.06	19.39	Indole-3-acetaldehyde;
ko00590	Arachidonic acid metabolism	1	.05	.06	20.94	Prostaglandin H2;
ko00230	Purine metabolism	1	.06	.06	16.70	Inosine

KEGG = Kyoto encyclopedia of genes and genes.

Combined with the above results, Theophylline (pos_906) and Inosine (pos_1800) in the drug resistant and drug sensitive groups were higher than that in the healthy control and latent infection groups (Fig. 3).

### 3.6. Relationship between serum markers and disease progression

**Figure 5.**
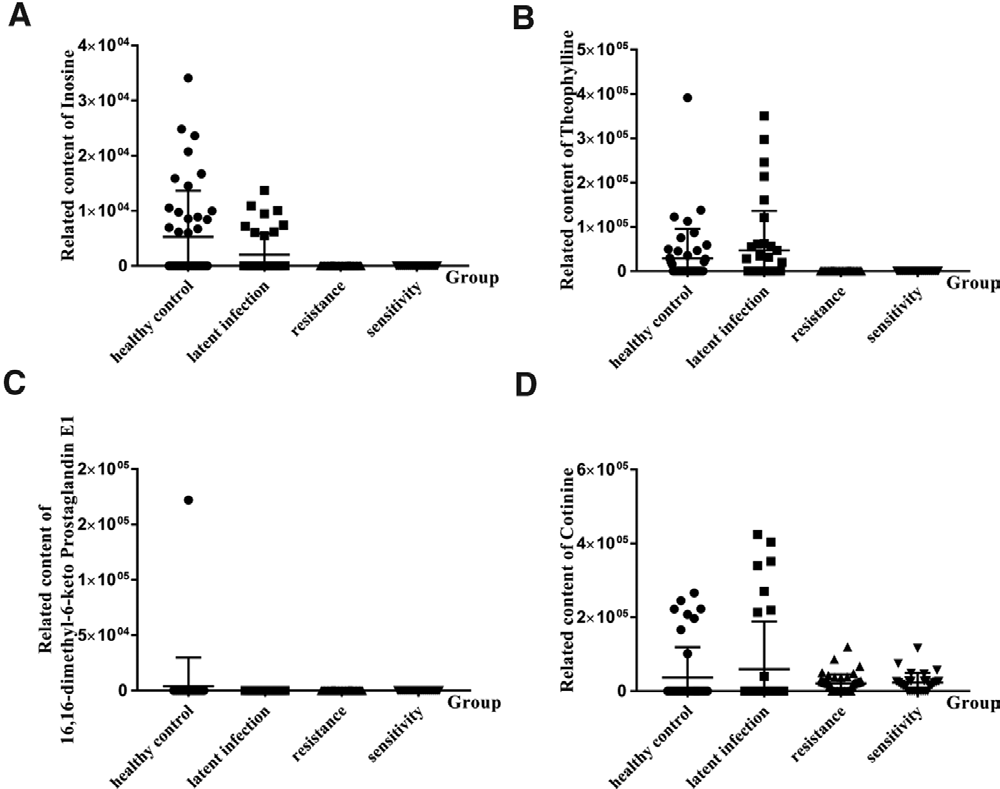
The relative contents of 4 potential serum markers. (A) Inosine; (B) Theophylline; (C) 16,16-dimethyl-6-keto Prostaglandin E1; (D) Cotinine.

Four potential serum markers were screened out using OPLS-DA, 1-way ANOVA, cluster analysis, and pathway analysis of differential metabolites. Therefore, the relative contents of these 4 serum markers (Inosine, 16,16-dimethyl-6-keto Prostaglandin E1, Theophylline, and Cotinine) in the 4 groups were further compared. As shown in Figure 5A and Figure 5C, the relative contents of Inosine and 16,16-dimethyl-6-keto Prostaglandin E1 were highest in the healthy control group, and Theophylline (Fig. 5B) and Cotinine (Fig. 5D) were highest in the latent infection group, implying that these metabolites might be used as biomarkers for the diagnosis of latent infection. Furthermore, the relative contents of Cotinine changed during the disease progression from healthy population to LTBI and then to active TB including drug-resistant TB infection and sensitive TB infection, implying that it can be used as potential markers to monitor disease progression and provide important ideas for timely disease control (Fig. 5D).

## 4. Discussion and conclusions

Tuberculosis causes a series of immunological reactions that can lead to mortality in the host, making it a major global public health concern. However, there are still a number of limitations to tuberculosis detection and treatment methods, including time, cost, and a high workload. This is also the reason why tuberculosis is so difficult to eradicate. In the fight against tuberculosis, the progress of metabolomics technology looks to give promise. Serum metabolites are a direct reflection of human biological activities. Distinct types of metabolites and their quantities usually signal different information, and this feedback is precise and quick. At the moment, research on metabolic indicators of Mycobacterium tuberculosis and tuberculosis is gaining popularity.^[17–19]^ Weiner J *et al*^[20]^ studied the metabolic patterns of tuberculosis patients (TB+), healthy uninfected persons (TST-), and latent infection (TST+) individuals. The difference in serum small molecule abundance among the 3 groups was determined by comparing 3 groups. There were significant differences in 176 compounds between TB patients and 2 healthy groups (TST- and TST+). However, the main serum biomarkers for diagnosing LTBI and tracking progression from latent infection to active TB infection are still rarely reported, which seriously affects the treatment cycle and prognosis of patients with tuberculosis.

In this study, serum markers of metabolic markers were discovered in latent infection patients and active TB patients in Guangdong using LC-MS/MS technology. The primary biological pathways were examined using machine learning and birth analysis to construct a serum monitoring model for early identification of latent infection and active pulmonary tuberculosis. It is planned to screen acceptable markers to evaluate the matching monitoring indicators from latently infected people to active tuberculosis infection, as well as give clinical guidelines for tuberculosis early detection, diagnosis, and treatment precision.^[21]^

Weiner *et al*^[20]^ investigated approximately 400 small molecules metabolites in the serum of uninfected people, latently infected healthy people, and patients with active tuberculosis in 2012. The results showed that the TB active group had lower levels of histidine, cysteine, glutamine, tryptophan, citrulline, and creatine than the 2 control groups, and that other identified compounds with markedly different abundance between the latently infected group and the TB active group were sialic acid (N-acetylneuraminate), 3-carboxy-4-methyl-5-propyl-2-furanpropanoic acid (CMPF), inosine, This does not entirely match our findings. Our findings demonstrated that the latently infected group had the lowest relative amount of Inosine, while other metabolites were inconsistent. The ethnic differences and dietary habits of clinical samples could be the cause. Furthermore, our findings revealed that the relative levels of Inosine and 16,16-dimethyl-6-keto Prostaglandin E1 were lowest in the latent infection group, implying that these molecules might be used as biomarkers to diagnose latent infection. Furthermore, the relative contents of Theophylline and Cotinine increased in the healthy control, latent infections, drug resistant, and drug sensitive groups, implying that these 2 metabolites can be used as potential markers to monitor disease progression and provide important ideas for timely disease control. Purine metabolism (Inosine), Arachidonic acid metabolism, Serotonergic synapse, Oxytocin signaling route, Platelet activation, Retrograde endocannabinoid signaling (Prostaglandin H2), and Caffeine metabolism were all annotated to the KEGG pathway (Theophylline). Our study has some limitations that should be mentioned. First, the sample size was relatively small and needs to be increased in further studies. Furthermore, this was a 2-center study, and multi-center study is needed in the future to support our conclusions.

In conclusion, this study screened and identified Inosine, 16,16-dimethyl-6-keto Prostaglandin E1, Theophylline, and Cotinine as potential serum biomarkers for diagnosing latent TB infection by LC-MS technique. Meanwhile, Cotinine have also been identified as potential biomarkers for monitoring disease progression. This strategy improves the sensitivity and specificity of disease diagnosis by combining small-molecule metabolic markers, providing broad clinical application prospects for disease assessment.

## Acknowledgements

We thank all the patients and healthy individuals for their participation.

## Author contributions

**Conceptualization:** Yan-Xia Li.

**Data curation:** Yu Duan.

**Formal analysis:** Yu-Qun Tang.

**Formal analysis:** Yu-Qun Tang.

**Investigation:** Kang-Di Zheng.

**Methodology:** Hua-Juan Liu.

**Supervision:** Dong-Zi Lin, Zhao Zhang.

**Validation:** Jun Wu.

**Writing-original draft:** Yan-Xia Li, Kang-Di Zheng.

**Writing-review & editing:** Zhao Zhang.

## Supplementary Material


