# Supplementary material for: Mass spectrometry-based identification of new serum biomarkers in patients with latent infection pulmonary tuberculosis
Source: Medicine (Baltimore). 2022 Dec 2;101(48):e32153. doi: 10.1097/MD.0000000000032153 (PMC9726300; doi:10.1097/MD.0000000000032153)
Supplement: Supplementary file 1 [file medi-101-e32153-s001.pdf]

Table S1 Screening results of differential metabolites among groups (partial results)

Metabolite	Mean				*p*
	Heal	Lat	Res	Sen	
Betaine	3.94	4.03	6.61	6.91	1.38E-25
4-Hydroxybenzaldehyde	-1.39	-1.57	-0.66	-0.23	2.79E-14
(R)-(+)-2-Pyrrolidone-5-carboxylic acid	2.89	3.07	-0.66	-0.23	3.31E-25
PHENACYLAMINE	0.14	0.00	-0.66	-0.23	6.29E-12
Hypoxanthine	0.30	0.11	0.51	0.59	0.00378
4-formyl Indole	-1.80	-1.94	-0.66	-0.23	1.10E-19
Coumarin	-2.54	-2.82	-0.66	-0.23	1.59E-24
L-Lysine	-2.29	-2.65	-0.66	-0.23	1.12E-23
2-Hydroxycinnamic acid	0.14	0.06	-0.66	-0.23	3.75E-13
Val Gly	-1.62	-2.02	-0.66	-0.23	2.55E-17
Theophylline	-2.76	-2.57	-0.66	-0.23	1.29E-18
butamben	1.33	1.21	-0.66	-0.23	5.09E-26
p-CHLOROPHENYLALANINE	0.78	0.87	-0.66	-0.23	2.12E-25
Acetylcarnitine	0.55	0.57	3.68	3.74	4.50E-24
DL-Tryptophan	3.05	3.04	-0.66	-0.23	2.79E-25
Pro Leu	-1.78	-1.87	-0.66	-0.23	7.77E-19
Inosine	-3.95	-6.22	-0.66	-0.23	1.03E-24
Phe Phe	1.32	1.01	-0.66	-0.23	4.11E-23
Leu Leu Phe	-1.24	-1.36	-0.66	-0.23	2.80E-11
Palmitoyl-L-carnitine	-1.45	-1.59	-0.66	-0.23	1.27E-14

11β-PGF2α Ethanolamide	-0.11	-0.21	-0.66	-0.23	5.84E-09
PC(16:0/0:0)[U] / PC(16:0/0:0)[rac]	6.46	6.38	-0.66	-0.23	1.13E-20
1-heptadecanoyl-sn-glycero-3-phosphocholine	-0.14	-0.36	-0.66	-0.23	9.57E-05
Tyr Arg Leu Ile Val	-1.25	-3.35	-0.66	-0.23	6.60E-19
w/o MS2:δ-Valerolactam	-0.15	0.29	-0.66	-0.23	0.00847
w/o MS2:Hydroxyhydroquinone	-1.98	-1.09	-0.66	-0.23	6.42E-15
w/o MS2:CYCLOCREATINE	-2.69	-3.05	-0.66	-0.23	1.45E-24
w/o MS2:2-Aminopropiophenone	-2.32	-2.29	-0.66	-0.23	2.91E-23
w/o MS2:Pyroglutamic acid	-1.69	-1.69	-0.29	0.51	3.22E-18
w/o MS2:N-HYDROXYMETHYLNICOTINAMIDE	-2.07	-2.03	-0.66	-0.23	3.76E-20
w/o MS2:Mechlorethamine	-2.30	-2.49	-0.66	-0.23	1.62E-24
w/o MS2:1-Benzylimidazole	-2.06	-2.34	-0.66	-0.23	1.50E-23
w/o MS2:3-[Bis(2-hydroxyethyl)amino]propanenitrile	-2.57	-2.53	-0.66	-0.23	5.18E-24
w/o MS2:Indoleacetaldehyde	-4.20	-5.67	-0.66	-0.23	1.24E-24
w/o MS2:3-thio-Pheneacrylic Acid methyl ester	1.61	1.67	-0.66	-0.23	5.11E-26
w/o MS2:Cotinine	-2.49	-2.23	2.55	2.85	2.29E-21
w/o MS2:3-Methylethcathinone	-4.17	-3.67	-0.66	-0.23	5.97E-25
w/o MS2:Thiabendazole	-0.84	-0.83	-0.66	-0.23	5.37E-10
w/o MS2:Kynurenine	-2.26	-2.66	-0.66	-0.23	3.17E-24
w/o MS2:PROPOXUR	-4.33	-6.90	-0.66	-0.23	1.42E-24

w/o MS2:Tuckolide; Decarestrictine D	-2.31	-2.78	-0.66	-0.23	2.19E-23
w/o MS2:5-Hydroxy-4-[3-(2-hydroxy-2-propanyl)-2-oxiranyl]-1-methyl-7-oxabicyclo[4.1.0]hept-3-en-2-one	-1.45	-1.69	-0.66	-0.23	1.33E-14
w/o MS2:Melatonin	-2.66	-3.27	-0.66	-0.23	3.70E-26
w/o MS2:3,4,5-Trimethoxycinnamic acid	-2.35	-2.94	-0.66	-0.23	2.13E-22
w/o MS2:Ribothymidine	-0.40	-0.38	-0.66	-0.23	0.00216
w/o MS2:Phe Ile	-3.01	-3.80	-0.66	1.04	8.36E-25
w/o MS2:Phe Asp	-2.82	-3.58	-0.66	-0.23	1.14E-24
w/o MS2:1-Methyladenosine	-2.37	-2.78	-0.66	-0.23	3.16E-25
w/o MS2:AG-17	-1.65	-3.18	-0.66	-0.23	1.71E-23
w/o MS2:(±)-Octanoylcarnitine	-2.73	-1.95	-0.66	-0.23	1.20E-20
w/o MS2:4-(1-Acetyloxypropen-2-yl-)-2-methoxyphenylisobutyrat; 4-(1-Acetoxy-2-propen-1-yl)-2-methoxyphenyl 2-methylpropanoate	-3.43	-4.72	-0.66	-0.23	2.20E-25
w/o MS2:Ser Lys Ser	-0.38	-0.54	-0.66	-0.23	3.23E-06
w/o MS2:PGH2	-3.93	-6.90	-0.66	-0.23	3.52E-24
w/o MS2:Trimethylolpropane trimethacrylate	-1.68	0.00	-0.66	-0.23	1.39E-10
w/o MS2:Lisuride	-4.18	-5.54	-0.66	-0.23	1.41E-24
w/o MS2:N-Oleoyl Glycine	-1.60	-1.46	0.84	-0.23	9.58E-18
w/o MS2:Ala Met Lys	-2.63	-6.90	-0.66	-0.23	2.14E-23

w/o MS2:Spiromesifen	-3.80	-5.71	-0.66	-0.23	1.01E-24
w/o MS2:13,14-dihydro-19(R)-hydroxy PGE1	-1.00	-1.90	-0.66	-0.23	4.89E-15
w/o MS2:16,16-dimethyl-6-keto Prostaglandin E1	-4.05	-6.90	-0.66	-0.23	2.59E-24
w/o MS2:Pro Arg Ile	-0.46	-1.20	-0.66	-0.23	3.97E-08
w/o MS2:Asn Phe Ile	-2.50	-6.90	-0.66	-0.23	2.14E-23
w/o MS2:Arg Gly Tyr	-3.70	-4.83	-0.66	-0.23	1.27E-24
Unknown	1.35	0.50	-0.66	-0.23	2.76E-19
w/o MS2:Myriocin	-1.01	-1.78	-0.66	-0.23	1.25E-14
w/o MS2:His Lys Met	-4.37	-6.08	-0.66	-0.23	1.46E-24
w/o MS2:Ala Asn Val Asp	-3.38	-4.23	-0.66	-0.19	1.17E-24
w/o MS2:Phe Leu Arg	-0.58	-1.07	-0.66	-0.23	1.44E-08
w/o MS2:Arg Met Met	-3.93	-5.10	-0.66	-0.23	7.15E-24
w/o MS2:HC Toxin	-2.40	-6.90	-0.66	-0.23	2.14E-23
w/o MS2:PE(16:0/0:0)	-1.33	-1.75	-0.66	-0.23	1.64E-16
w/o MS2:Pristimerin	-0.73	-0.63	-0.66	-0.23	2.85E-10
w/o MS2:Buprenorphine	-0.22	-0.43	-0.66	-0.23	0.00079
w/o MS2:(3E)-7-Hydroxy-3,7-dimethyl-3-octen-1-yl 6-O-(6-deoxy-?-L-mannopyranosyl)-?-D-glucopyranoside	-2.51	-6.90	-0.66	-0.23	2.14E-23
w/o MS2:PG(18:1(9Z)/0:0)	-3.30	-5.33	-0.66	-0.23	7.09E-25
w/o MS2:Arg Thr Asp Arg	-2.93	-3.16	-0.66	-0.23	1.38E-24
w/o MS2:Phe Glu Ser Phe Gly	-4.37	-4.19	-0.66	-0.23	1.83E-25

w/o MS2:Leu Leu Asp Leu Leu	3.21	3.38	-0.66	-0.23	2.35E-26
w/o MS2:Arjunglucoside II; (2S,3R,4S,5S,6R)-3,4,5-trihydroxy-6-(hydroxymethyl)tetrahydro-2H-pyran-2-yl (4aS,6aS,6bR,9R,10R,11R,12aR)-10,11-dihydroxy-9-(hydroxymethyl)-2,2,6a,6b,9,12a-hexamethyl-1,3,4,5,6,6a,6b,7,8,8a,9,10,11,12,12a,12b,13,14b-octadecahydropicene-4a(2H)-carboxylate	-3.52	-4.46	-0.66	-0.23	2.35E-23

Heal means healthy control, Lat means latent infection, Res means resistance, and Sen means sensitivity group.
